# Supplementary material for: The causal role of circulating inflammatory markers in osteoporosis: a bidirectional Mendelian randomized study
Source: Front Immunol. 2024 Jul 18;15:1412298. doi: 10.3389/fimmu.2024.1412298 (PMC11291241; doi:10.3389/fimmu.2024.1412298)
Supplement: Supplementary file 1 [file DataSheet_1.zip › supplementary material/OSTEOPOROSIS-CIM/GCST90274770.tsv.gz_forest.pdf]

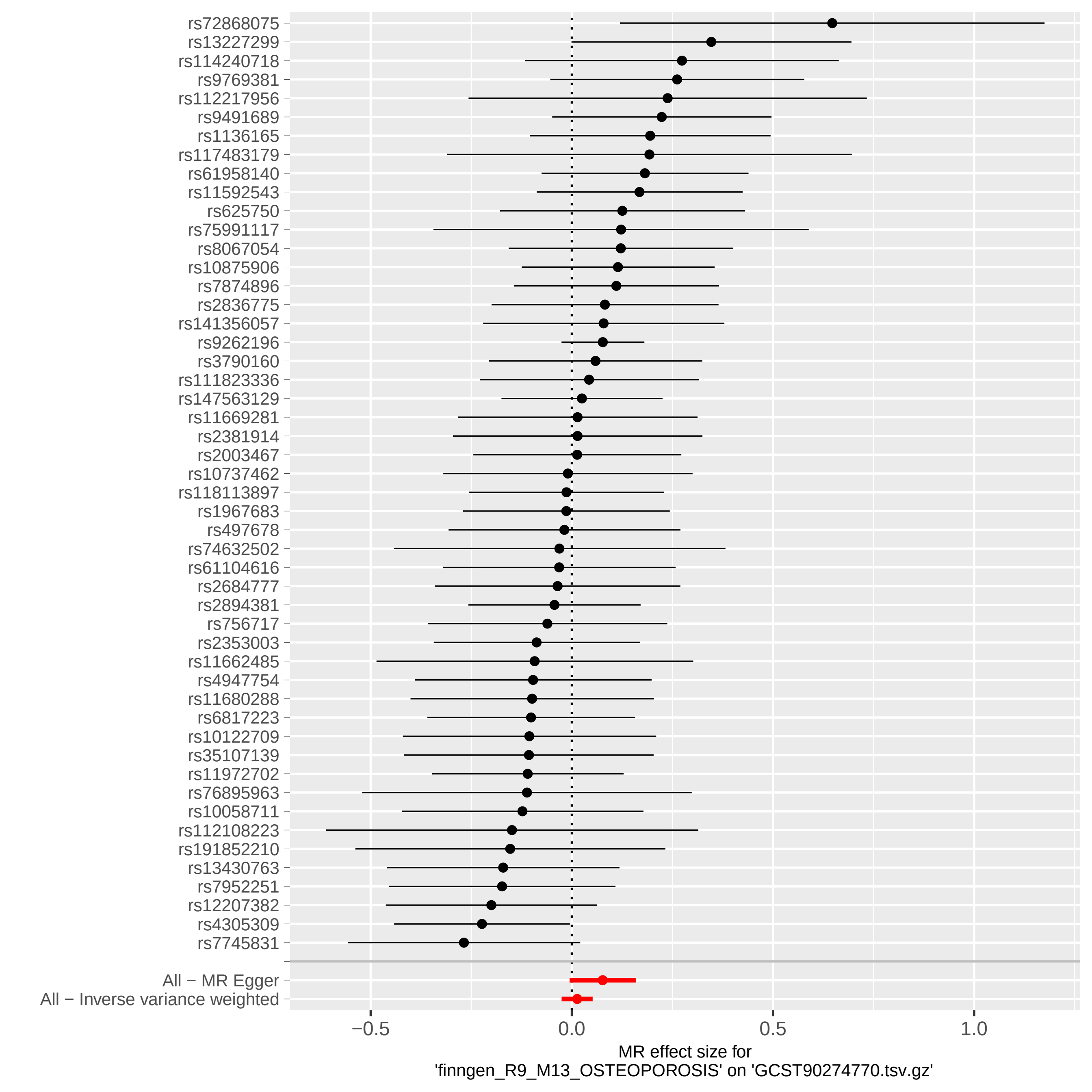
rs72868075
rs13227299
rs114240718
rs9769381
rs112217956
rs9491689
rs1136165
rs117483179
rs61958140
rs11592543
rs625750
rs75991117
rs8067054
rs10875906
rs7874896
rs2836775
rs141356057
rs9262196
rs3790160
rs111823336
rs147563129
rs11669281
rs2381914
rs2003467
rs10737462
rs118113897
rs1967683
rs497678
rs74632502
rs61104616
rs2684777
rs2894381
rs756717
rs2353003
rs11662485
rs4947754
rs11680288
rs6817223
rs10122709
rs35107139
rs11972702
rs76895963
rs10058711
rs112108223
rs191852210
rs13430763
rs7952251
rs12207382
rs4305309
rs7745831
All − MR Egger
All − Inverse variance weighted
−0.5
0.0
0.5
1.0
MR effect size for
'finngen_R9_M13_OSTEOPOROSIS' on 'GCST90274770.tsv.gz'
